# The genetic alteration spectrum of the SWI/SNF complex: The oncogenic roles of BRD9 and ACTL6A

**DOI:** 10.1371/journal.pone.0222305

**Published:** 2019-09-10

**Authors:** Xiaoxian Sima, Jiangnan He, Jie Peng, Yanmei Xu, Feng Zhang, Libin Deng

**Affiliations:** 1 Queen Mary College, Nanchang University, Nanchang, Jiangxi, P.R. China; 2 Jiangxi Provincial Key Laboratory of Preventive Medicine, School of Public Health, Nanchang University, Nanchang, Jiangxi, P.R. China; 3 The Second Affiliated Hospital of Nanchang University, Nanchang, Jiangxi, P.R. China; 4 Institute of Translational Medicine, Nanchang University, Nanchang, Jiangxi, P. R. China; 5 College of Basic Medical Science, Nanchang University, Nanchang, Jiangxi, P.R. China; Massachusetts Eye and Ear Infirmary, Harvard Medical School, UNITED STATES

## Abstract

SWItch/Sucrose NonFermentable (SWI/SNF) is a set of multi-subunits chromatin remodeling complexes, playing important roles in a variety of biological processes. Loss-of-function mutations in the genes encoding SWI/SNF subunits have been reported in more than 20% of human cancers. Thus, it was widely considered as a tumor suppressor in the past decade. However, recent studies reported that some genes encoding subunits of SWI/SNF complexes were amplified and play oncogenic roles in human cancers. In present study, we summarized the genetic alteration spectrum of SWI/SNF complexes, and firstly systematically estimated both the copy number variations and point mutations of all 30 genes encoding the subunits in this complex. Additionally, the bioinformatics analyses were performed for two significantly amplified genes, *ACTL6A* and *BRD9*, to investigate their oncogenic roles in human cancers. Our findings may lay a foundation for the discovery of potential treatment targets in SWI/SNF complexes of cancers.

## Introduction

**Fig 1:**
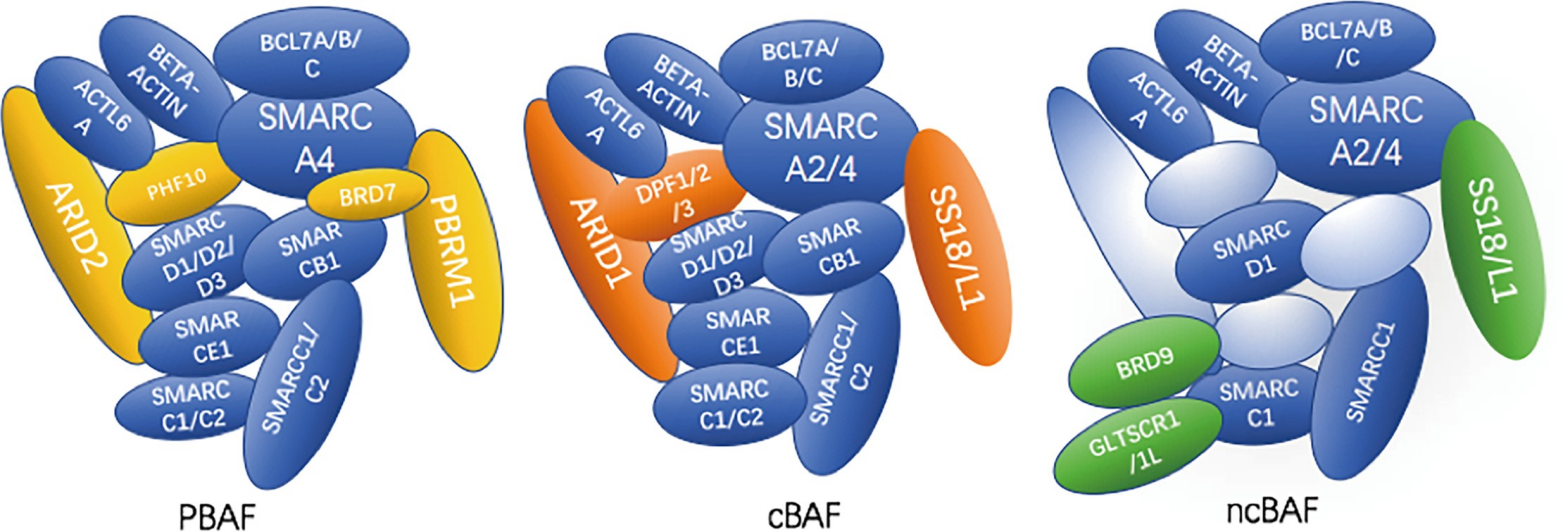
SWI/SNF complex structures. Schematic representation of canonical BAF, PBAF and noncanonical BAF composition. The orange subunits are unique to cBAF, yellow subunits are unique to PBAF, and green subunits are unique to ncBAF.

SWItch/Sucrose NonFermentable (SWI/SNF) is a set of highly conserved multisubunit complexes [1]. These complexes use the energy from adenosine triphosphate (ATP) hydrolysis to remodel specific nucleosomes in the whole genome [1]. These SWI/SWF complexes could be classified into three categories: canonical BAF (cBAF), PBAF and noncanonical BAF complex (ncBAF or GBAF) [2]. In mammals, each SWI/SNF complex is assembled by at least ten subunits encoded by total 30 genes [3]. The combinations of these subunits are highly variable, and some subunits are unique to specific complexes. For example, ARID1A, ARID1B, and DPF2 are specifically in cBAF; PBRM1, ARID2, and BRD7 are uniquely in PBAF; and GLTSCR1/GLTSCR1L and BRD9 exist in only ncBAF complexes [4] (Fig 1).

SWI/SNF complexes play a number of critical roles in a series of physiological processes and in the development of diseases, especially in cancers [5]. In the past decade, SWI/SWF has been known as one of the most frequently mutated chromatin regulators in human cancers [6]. Loss-of-function mutations in the genes encoding SWI/SNF subunits are reported in more than 20% of human cancers, and the critical tumor suppressive role of the SWI/SNF complex has been widely acknowledged [7]. However, with more new subunits being discovered, recent studies based on high-throughput technology have noted that some subunits were amplified in cancer cells [2]. For example, a scaffolding subunit, ACTL6A, was amplified and functioned as an oncogenic driver in squamous cell carcinoma [8]. These results suggested that not all of the genes encoding the subunits of the SWI/SNF complex have loss-of-function mutations in cancer. Some amplified genes of SWI/SNF complexes might play oncogenic roles in tumor formation. Moreover, the amplified subunits could be potential treatment targets, which could be inhibited by small-molecule inhibitors.

Therefore, to understand which genes are oncogenic and find some potential treatment targets in the SWI/SNF complex, a systematic spectrum of genetic alterations was explored based on the latest structural study and high-throughput sequencing research on SWI/SNF complexes. Moreover, pathway enrichment analyses were also performed for *ACTL6A* and *BRD9*, two noticeably amplified genes, to explain their oncogenic mechanisms.

## Methods and materials

### Genetic alteration data from TCGA

In this study, we collected datasets involving 25 cancers (cases >100 samples) across 10,931 patients from The Cancer Genome Atlas (TCGA) database via cBioPortal (http://www.cbioportal.org), searched up to February 16, 2019. The data, including the genetic alteration frequency of all 30 genes encoding the subunits of the SWI/SNF complex in each cancer type, were then reorganized and presented in a diagram.

### Sequencing data from GEO

The gene expression profiles for the *ACTL6A* or *BRD9* inhibited cell lines were acquired from the Gene Expression Omnibus (GEO) database (https://www.ncbi.nlm.nih.gov/geo/). The shACTL6A microarray data were downloaded from Rose et al. (GSE88831) based on the head and neck squamous cell carcinoma (HNSCC) cell line FADU. The data were processed by GEO2R, and then a ranked gene list related to the inhibition of *ACTL6A* was obtained. The RNA-Seq data with *BRD9* inhibitor treatment were acquired from Hohmann AF. et al. (GSE79391), based on the HeLa cell line. In this experiment, the HeLa cell line was treated with BI-7273 (an inhibitor of *BRD9*) in the cell line. Afterwards, differentially expressed gene (DEG) analysis based on this profile was performed in a website (https://vimi01.shinyapps.io/edger/) to obtain a file including a ranked genes list for *BRD9*. Moreover, to make the result more convincing, three additional gene expression datasets (GSE120235, GSE129437 and GSE79284) for BRD9 KD based on 4 different tumor cell lines (G401, MV4-11, HL-60, RN2) were also download, and the following analysis were also performed next.

### Pathway analysis

WebGestalt (WEB-based GEneSeT AnaLysis Toolkit) (http://www.webgestalt.org) is a large-scale integrated data analysis website that provides comprehensive enrichment analyses. KEGG pathway databases from MSigDB (http://software.broadinstitute.org/gsea/msigdb) were used to analyze the biological pathways and functions of the genes of interest. The ranked gene list of *BRD9* and *ACTL6A* were separately uploaded and regarded as experimental groups, choosing genome as a control group in reference gene list. The significantly enriched biological pathways were screened based on a false discovery rate (FDR) < 0.5. Then, the two most highly associated pathways, the oxidative phosphorylation pathway and the ribosome pathway, were selected for GSEA.

### Gene set enrichment analysis (GSEA) and TCGA data analysis

Next, the two most highly associated pathways, the oxidative phosphorylation pathway and the ribosome pathway, were selected for GSEA, which is supported by the Broad Institute website (http://www.broadinstitute.org/gsea/index.jsp). The ranked gene lists of enriched pathways were used to perform the GSEA analysis based on cell- experiment data. Additionally, based on the clinical data across 31 cancer types, the mRNA expression levels of these involved genes of oxidative phosphorylation pathways and ribosomal pathways were checked in GEPIA (http://gepia.cancer-pku.cn/detail), a website provided the visualized date based on TCGA database.

### Statistical analysis

The mRNA expression level data of *ACTL6A* and *BRD9* and survival data were also obtained from cBioPortal (http://www.cbioportal.org). The relationship between the mRNA expression level and copy number variation of *ACTL6A* or *BRD9* was described by boxplots using TIMER (http://cistrome.dfci.harvard.edu/TIMER/). This was calculated based on the Wilcoxon test method. Besides, we have researched the protein expression levels of *BRD9* and *ACTL6A* in the human protein atlas (https://www.proteinatlas.org), a website provided the expression information of 24,000 proteins distributed in both normal tissues and cancers based on immunofluorescence method. The protein expression levels of ACTL6A and BRD9 in 17 cancers were checked and compared to their expression levels in the corresponding normal tissues. And these amounts of sections were implemented chi-square test to verify the significance. Moreover, the Cox regression model of SPSS software was used to analyze the survival data and calculate the specific hazard ratio (HR) and standard error (SE) of each cancer type for *ACTL6A* and *BRD9*. Then, by combining the survival data and mRNA expression level data using a fixed effects model in Review Manager 5.3 software, a pooled HR and SE was obtained, and the associated relationships between *ACTL6A* and *BRD9* mRNA expression level and the survival rate of the samples were observed Statistical heterogeneity of studies was measured by Chi-square based Q test and *I*^2^-square. If the *I*^2^≥50% *and P*≤0.01, these HR and CI would be pooled by using the random-effect model. If *I*^2^ *and P value* are not suitable the former condition, then pooled data should apply fixed-effect model.

## Results

### Different cancers have distinct types of genetic alterations in the SWI/SNF complexes

**Fig 2:**
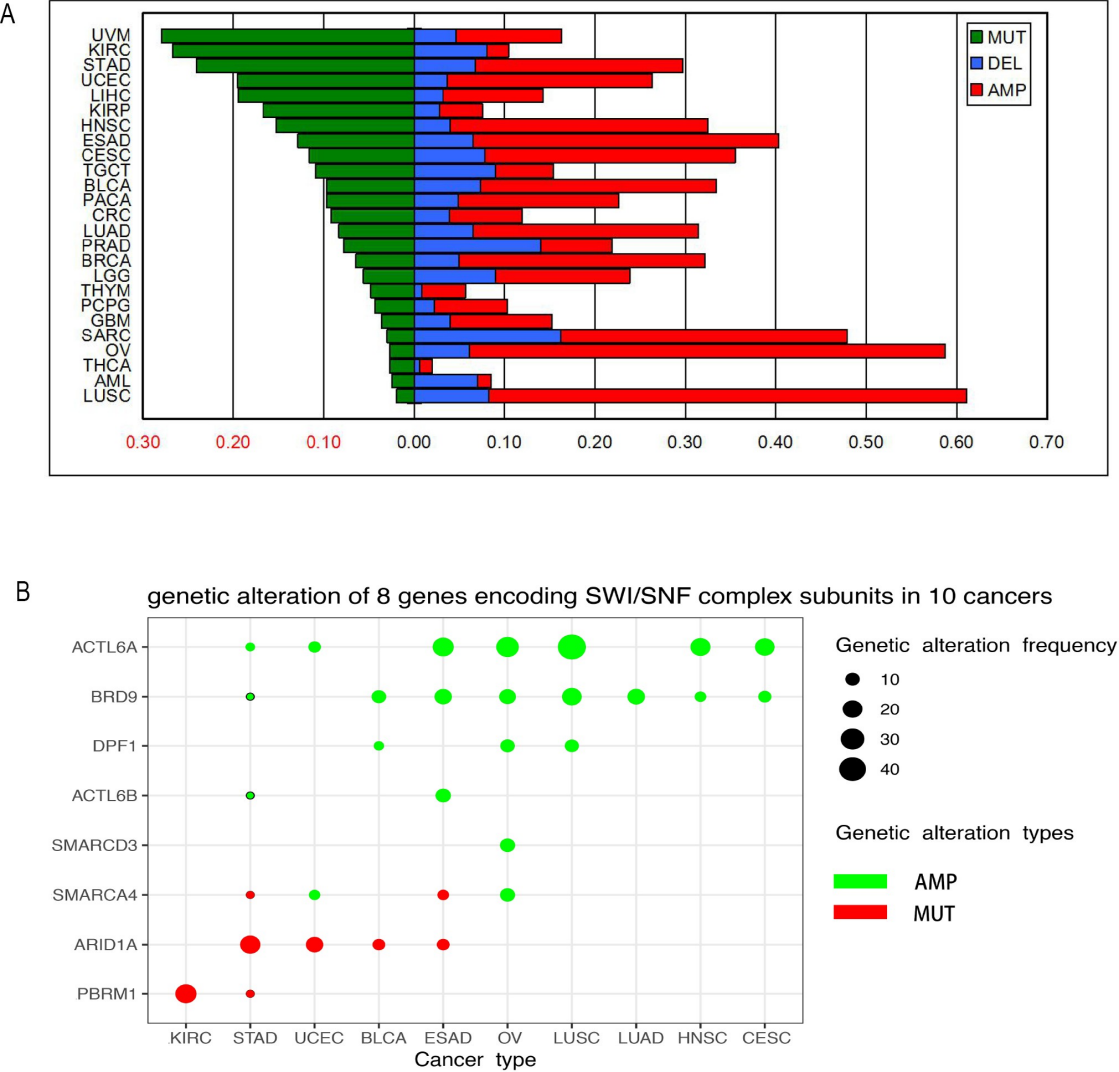
SWI/SNF complex mutations are quite pervasive within a wide range of human cancers. (A) This bar chart shows the genetic alteration frequency of the SWI/SNF complex within 25 types of cancers. Green indicates point mutations, blue indicates deletions, and red indicates amplifications. It is clear that the copy number variation (CNV) frequency of the SWI/SNF complex within 25 types of cancers constitutes a large proportion of the chart. There are 14 cancers in which the CNV frequency of the SWI/SNF complex exceeds 20%. However, for point mutations, there are only 3 types of cancers (melanoma, ccRCC, and stomach cancer) with frequencies above 20%. (B) This figure shows the genetic alteration frequency of 8 subunits among 10 cancer types. Red indicates point mutations, and green indicates CNVs. The differing sizes of the bubbles represent the genetic alteration frequency of each subunit among 10 cancer types. Larger bubbles indicate a higher genetic alteration frequency of subunits within these cancer types.

The total genetic alteration rate of the SWI/SNF complex is approximately 43% (4,587/10,931), which again emphasized that the genes encoding subunits of SWI/SNF complexes are commonly mutated in human cancers. These genetic alterations were divided into 4 groups: the point mutation group (n = 1,309, 11.98%), the amplification group (n = 2,343, 21.43%), the deletion group (n = 684, 6.26%), and the multiple alteration group (n = 514, 4.95%). The genetic alteration situation of these 4 groups in 25 cancer types were shown in the S1 Fig. As shown in Fig 2, SWI/SNF complex genes tended to have point mutations in some cancers, such as melanoma (26.97%), clear cell renal cell carcinoma (ccRCC) (26.77%) and stomach cancer (24.06%). However, there are a variety of cancer types in which the genetic alterations of the SWI/SNF complex tended to have copy number variations, particularly amplifications, such as in lung squamous cell carcinoma (61.06%), ovarian cancer (58.71%), sarcoma (47.89%), esophageal cancer (40.32%), cervical cancer (35.48%), head and neck cancer (32.45%), breast cancer (32.13%), bladder cancer (33.41%), lung adenocarcinoma (31.39%), stomach cancer (29.70%), uterine cancer (26.28%), glioma (23.87%), liver cancer (23.08%) and prostate cancer (22.58%) (Fig 2A). These results suggested that the amplification of distinct genes was the main alteration type in most cancers, and the subunits of SWI/SNF complexes may play an essential oncogenic role in cancers.

Moreover, we detected the genetic alterations of each subunit of the SWI/SNF complex. Different types of genetic alterations occurred more frequently in some subunits within the SWI/SNF complex in some cancers. The point mutation rate and amplification rate of each gene in 25 cancers were also listed in the S1 Table. The genes (mutation frequency >10% in at least one tumor) were shown in the bubble plot (Fig 2B). The most commonly mutated gene was *ARID1A*, which was highly mutated in uterine cancer (14.78%) and stomach cancer (20.92%), as previously reported. Additionally, *ACTL6A* and *BRD9* showed a border spectrum of genetic alterations in five different cancer types. *ACTL6A* was amplified in lung squamous cell carcinoma (44.42%), cervical cancer (19.03%), esophageal carcinoma (22.58%), head and neck cancer (20.19%), and ovarian cancer (26.73%). *BRD9* was amplified in bladder cancer (10.17%), lung squamous cell carcinoma (19.37%), lung adenocarcinoma (15.02%), esophageal carcinoma (14.52%) and ovarian cancer (13.53%). These results demonstrated that the different mutations of each subunit played distinct roles in different cancer types. In addition, the amplification of *ACTL6A* and *BRD9* commonly occurred in multiple cancers and may play crucial roles in tumor formation.

### *BRD9* and *ACTL6A* may act as oncogenic drivers in cancer development

**Fig 3:**
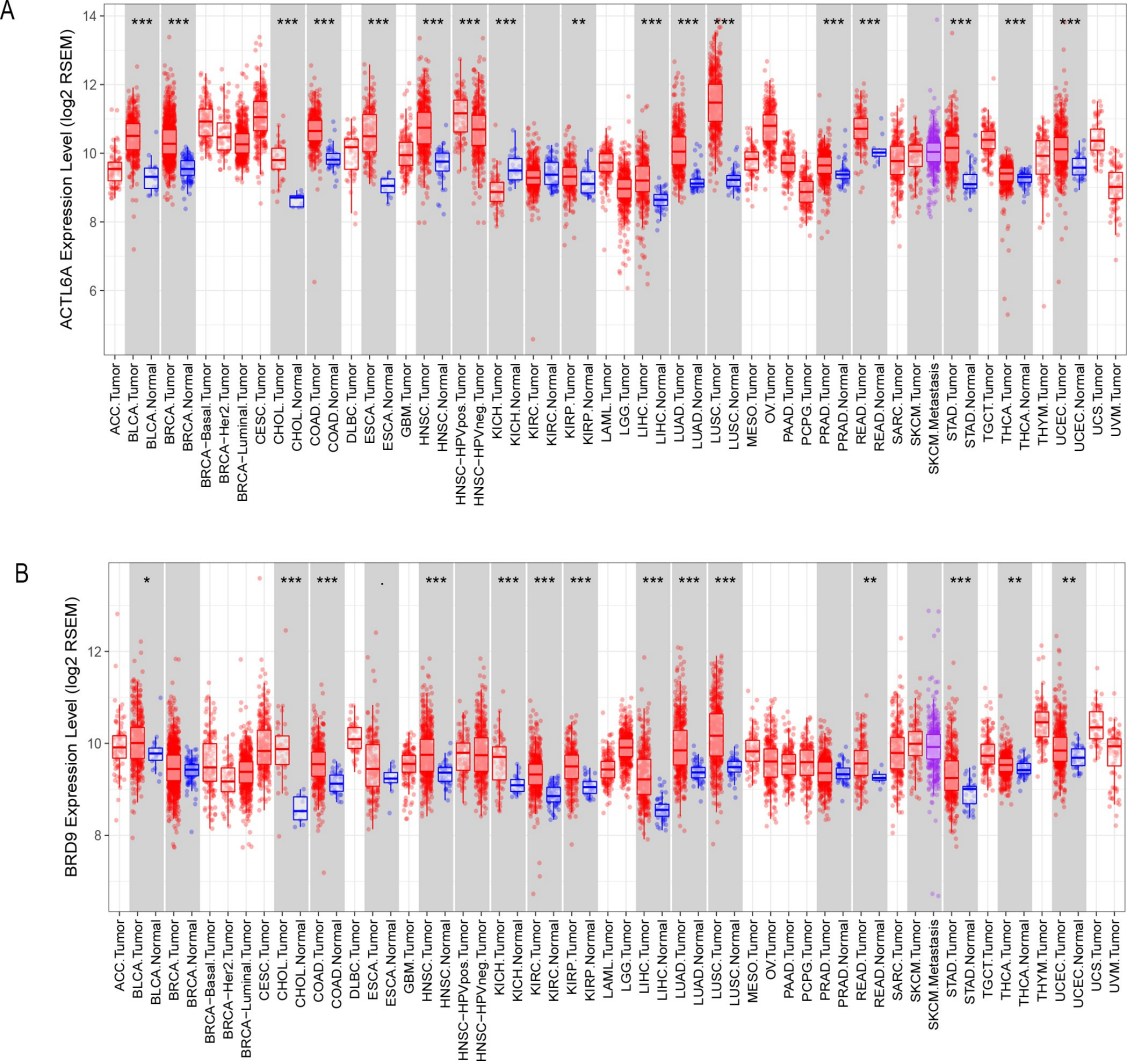
Comparison of *ACTL6A* and *BRD9* mRNA expression between the normal tissues and tumor tissues. A) ACTL6A differentially mRNA expression between some normal tissues and serval cancers. (B) BRD9 differentially mRNA expression between normal tissues and a wide range of cancers. (P value significant codes: 0≤***<0.001≤**<0.01≤*<0.05≤.<0.1). These boxplots show that the mRNA expression of *ACTL6A* and *BRD9* within most tumors are higher than that in normal cells.

To determine whether the increased copy number of *ACTL6A* and *BRD9* led to an increase in their expression levels, we acquired two pan-cancer boxplots (Fig 3) regarding the difference of these two genes expression between normal and tumor tissues from Tumor IMmune Estimation Resource (Timer). Fig 3 showed that in most cancers investigated, the higher copy number of *ACTL6A* or *BRD9* is, the higher *ACTL6A* or *BRD9* mRNA levels. Furthermore, the protein expression levels of ACTL6A and BRD9 in 17 cancers were checked and compared to the expression levels of these two genes in the corresponding normal tissues in HPA. The result shown that the expression levels of these two proteins are higher in some cancers than normal tissue. However, according to the chi-square test, we still can not make a conclusion that the protein levels are increased with the amplification of the copy number of *ACTL6A* or *BRD9* due to the limited sample size and qualitative data (p value > 0.05). The related result was shown in S2 Table.

**Fig 4:**
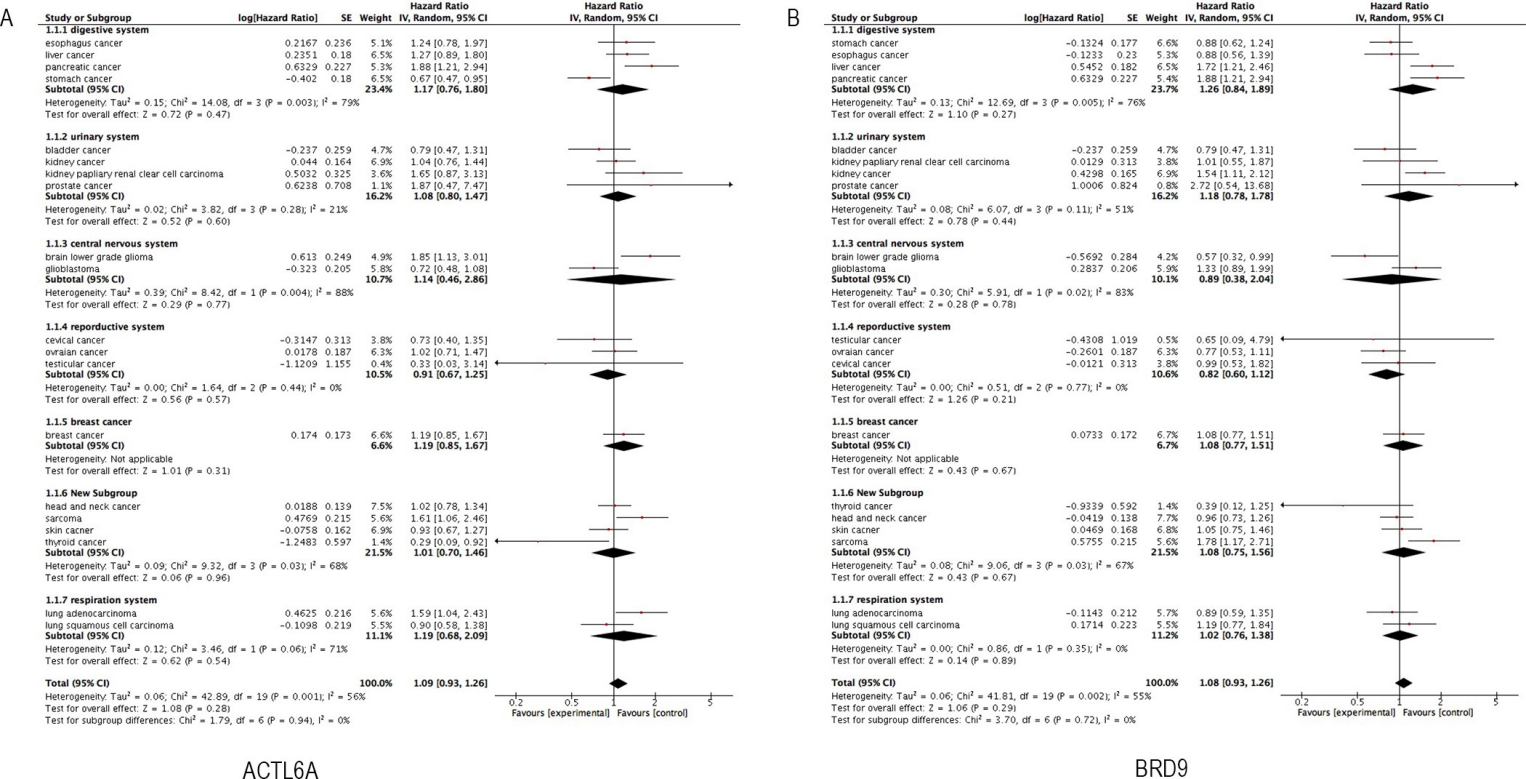
Relationship between *ACTL6A* and *BRD9* mRNA expression and survival rate within each patient from TCGA. (A) This forest plot shows the relationship between the *ACTL6A* mRNA expression level and the survival rate of cases. The combined HR is 1.09 (HR>1), 95% CI (0.93,1.26). (B) This forest plot represents the relationship between the *BRD9* mRNA expression level and survival rate of cases. The combined HR was 1.08 (HR>1), 95% CI (0.93, 1.26).

Apart from that, we obtained a total of 7,370 patients’ mRNA expression data within 19 different cancers from TCGA database as well (case > 100). These data were analyzed with the Cox model and random effects model, and two forest plots were constructed (Fig 4). Albeit overall trend that was more likely to affect survival time of cancer patient is not significant, we still spotted some specific cancers that were statistically significantly associated with them. Regarding *ACTL6A*, we found that *ACTL6A* expression might significantly affect the survival time of patients with pancreatic cancer (HR, 1.88; 95% CI, 1.21 to 2.94), brain lower grade glioma (HR, 1.85; 95% CI, 1.13 to 3.01), lung adenocarcinoma (HR, 1.59; 95% CI, 1.04 to 2.43), and sarcoma (HR, 1.61; 95% CI, 1.06 to 2.46) (Fig 4A). For *BRD9*, we discovered that the survival time of patients with 3 cancer types was significantly shorted by high mRNA expression of *BRD9*: liver cancer (HR, 1.72; 95% CI, 1.21 to 2.46), ccRCC (HR, 1.54; 95% CI, 1.11 to 2.12), and sarcoma (HR, 1.78; 95% CI, 1.17 to 2.71) (Fig 4B).

### Both ACTL6A and BRD9 significantly affect the oxidative phosphorylation pathways and ribosomal pathways

**Fig 5:**
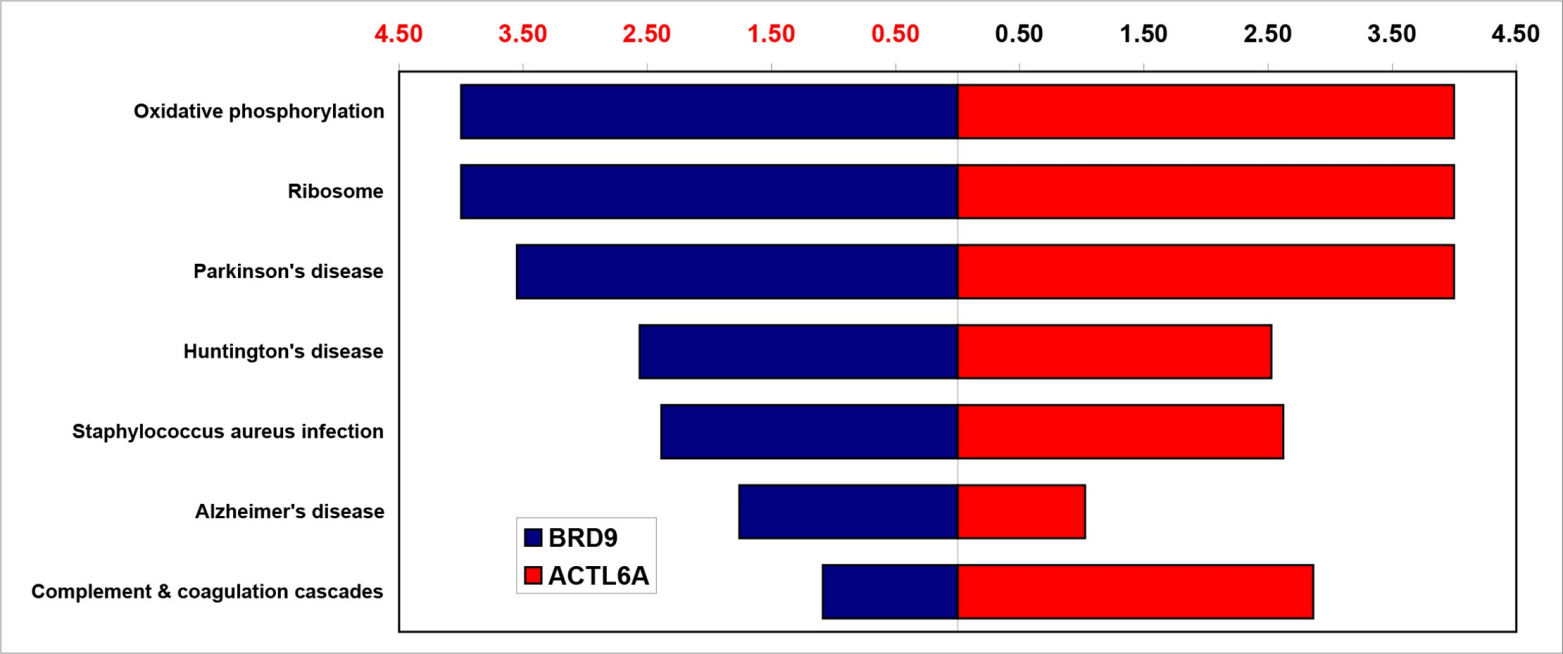
*ACTL6A* and *BRD9* statistically significantly associated biological pathways. It is a bar chart depicting the ACTL6A- and BRD9-associated pathways. The y-axis indicates the pathway name, and the x-axis indicates the enrichment factor. This chart shows that ACTL6A and BRD9 are most significantly associated with the oxidative phosphorylation and ribosome pathways.

**Fig 6:**
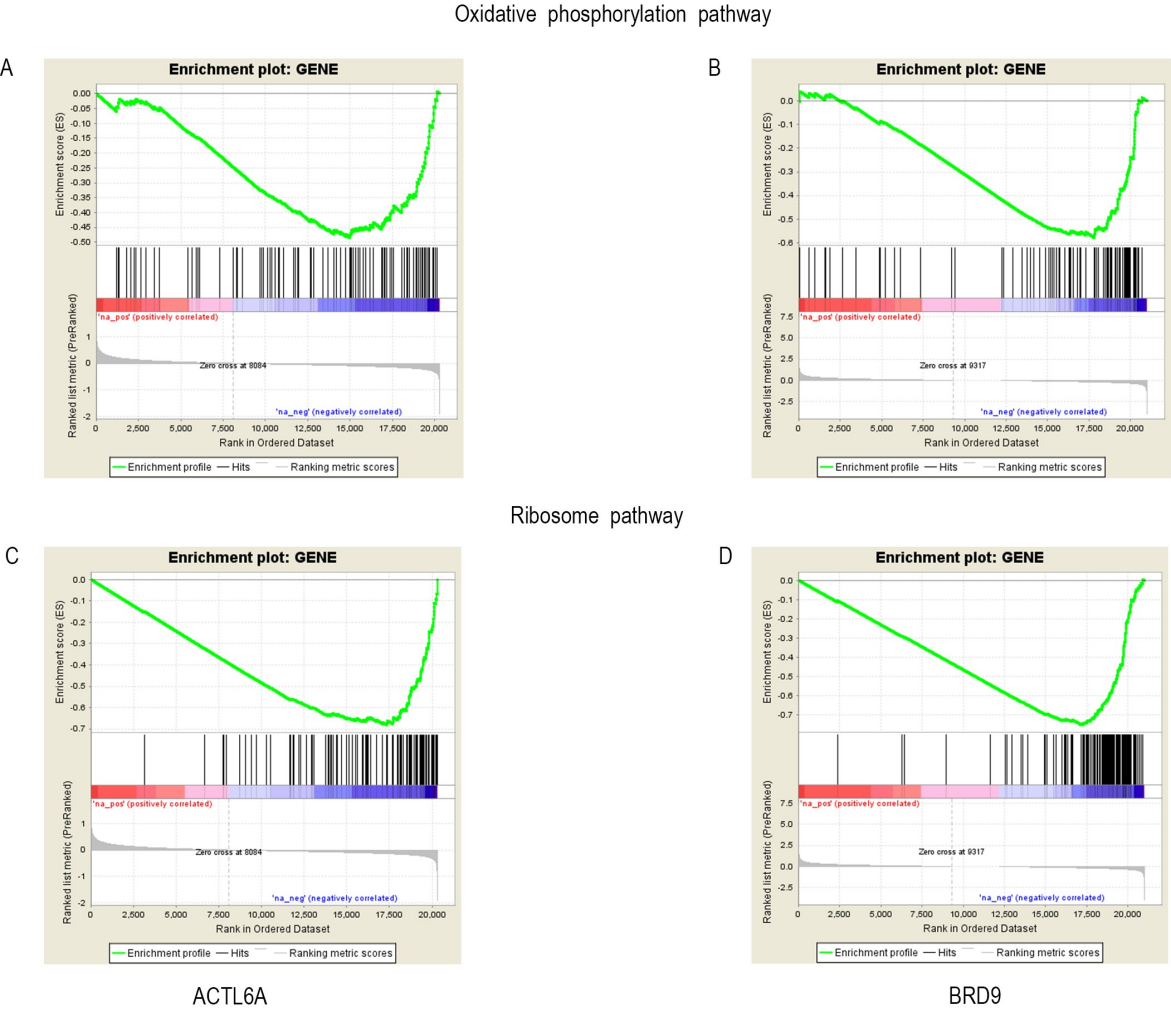
GSEA results of ACTL6A and BRD9 within oxidative phosphorylation and ribosome pathways. (A) Enrichment plot of the oxidative phosphorylation for *ACTL6A*. (B) Enrichment plot of the oxidative phosphorylation for *BRD9*. (C) Enrichment plot of ribosome pathways for *ACTL6A*. (D) Enrichment plot of ribosome pathways for *BRD9*. Among these figures, there are two phenotypes—the positive and the negative groups in each plot. The red bar marks the upregulated group. The blue bar indicates the downregulated group. Each vertical line of two plots represents the genes involved in oxidative phosphorylation. Their enrichment score (ES) is calculated following the list genes in oxidative phosphorylation. When encountering the involved genes, the ES will increase, and vice versa for the uninvolved genes. The trend lines of the two plots are downward following the gene list. Therefore, the involved genes are mostly clustered in the downregulated zone.

To study how the cancer cells were affected by the genetic alteration of *BRD9* or *ACTL6A*, we performed KEGG analysis and GSEA analysis. Ranked gene lists for *ACTL6A* and *BRD9* were separately uploaded to WebGestalt (http://www.webgestalt.org/). The results were shown in Fig 5. Surprisingly, both oxidative phosphorylation pathway and ribosome pathway were most statistically significantly associated with experiments involving the inhibition of *ACTL6A* and *BRD9*. These results indicated that the amplification of both *BRD9* and *ACTL6A* affect these essential biological pathways in human cancers. Besides, 30 overlapping differential expressed genes involved in these two pathways has been shown in the S3 Table. Then, GSEA was performed to understand how these two pathways were affected in the cell lines with inhibited *ACTL6A* or *BRD9* expression. The results were shown in Fig 6A–6D, most genes involved in oxidative phosphorylation and the ribosome pathways were downregulated in both experiments. We have also checked the mRNAs expression levels of genes of the oxidative phosphorylation and ribosome pathways in GEPIA based on TCGA datasets, the mRNA changes for the affected genes of these two pathways across 31 cancer types in a table in the S4 Table. These clinical data show that the genes involved in these two pathways were mostly up-regulated in most cancer types. Particularly, the genes of oxidative phosphorylation pathway were significantly overexpressed in DLBC, GBM, LIHC, PAAD, THYM. And for the genes involved in ribosomal pathway, they were remarkably overexpressed in DLBC, GBM, LGG, LIHC, PAAD, READ, TGCT, THYM. Differentially, we also found that larger proportion of these genes were down-regulated in Acute Myeloid Leukemia (LAML). Moreover, the results of KEGG analyses for additional 3 datasets for BRD9 knockdown were associated to oxidoreductase activity in hl60 cell line, ribosome biogenesis in RN2 cell line, and also related to the translation in G401 cell line. And the results were list in the S5 Table.

### The co-occurrence relationship among subunits within the SWI/SNF complex

**Fig 7:**
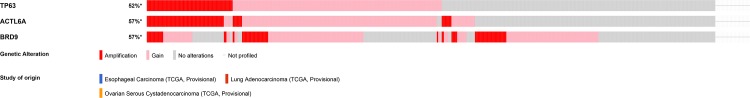
OncoPrint plot. This plot shows the genomic profile of the gain and amplification of *TP63*, *ACTL6A* and *BRD9* in cancer patients. These TCGA datasets from cBioPortal are based on the following 3 cancer types: ovarian cancer, esophageal cancer and lung adenocarcinoma. Each vertical line indicates one genetic file of the patient. The red bar indicates the amplification, the pink bar indicates the gain, and the gray bar indicates no alteration. *ACTL6A* is most significantly amplified with *TP63* (p<0.001, log odds ratio>3). *BRD9* is also significantly amplified with *ACTL6A* (p<0.001, log odds ratio = 1.051) and *TP63* (p<0.001, log odds ratio = 0.907).

Additionally, based on a wide variety of TCGA data, co-mutation relationships between these genes were discovered in the SWI/SNF complex, shown in S6 Table, indicating that the various functional collaborations of mutated genes may drive the oncogenic process. Within the 3 cancer types (ovarian cancer, esophageal cancer and lung adenocarcinoma) that were commonly mutated in *BRD9* and *ACTL6A*, a significant comutation relationship (p<0.001, log odds ratio = 1.051) was also shown in *BRD9* and *ACTL6A* (Fig 7), which suggested that they may drive the oncogenic process together by affecting the same pathways.

In addition, *ACTL6A* was co-amplified with *TP63*, which functions as a central oncogenic driver [9]. This result suggests that the amplification of *ACTL6A* and *TP63* could be a common oncogenic mechanism across human cancers. Fig 7 also indicates that *ACTL6A* was remarkably coamplified with *TP63* (p<0.001, log odds ratio>3) across ovarian cancer, esophageal cancer and lung adenocarcinoma. Moreover, *BRD9* showed significant coamplification with *TP63* (p<0.001 log odds ratio = 0.907). These results suggest that these 3 subunits may mediate a common pathway in tumor cells.

## Discussion

In the present study, we summarized the genetic alteration spectrum of SWI/SNF complexes and systematically estimated the copy number variations and point mutations of all 30 genes encoding the subunits in this complex. Our analysis was based on high-throughput sequencing data from 25 published research studies, representing 10,931 cases. Different tumors tended to have distinct genetic alteration types in specific genes. The most frequently mutated (point mutation) genes were *ARID1A*, *ARID1B*, *PBRM1*, and *SMARCA4*, which is consistent with the findings of a prior study.

Moreover, the copy number variations of these genes were first highlighted. Among them, *BRD9* and *ACTL6A* showed a high amplification frequency across multiple cancers. Although the result of immunofluorescence indicated that the protein levels are high in cancers, whether the protein levels are increased with the amplification of the copy number of *ACTL6A* or *BRD9* is still not clear (p value>0.05). Therefore, we consulted the literatures. Based on fluorescence in situ hybridization (FISH), a research indicated that the protein of BRD9 overexpressed in cervical cancer [10]. By using western blotting analysis, the BRD9 protein levels in CD34+ cell and some cancer cell lines (HL60, K562 and U937) were compared, another research represented that this protein was also elevated in leukemia and lymphoma [11]. For ACTL6A, western blotting analyzes indicated that high ACTL6A protein levels in colon tumor cells, liver tumor cells and glioma [12,13]. Therefore, at least in these cancer types, the protein levels of both BRD9 and ACTL6A are increased with the increase of mRNA, and there is no any altered translocation has been discovered so far.

In addition, based on the clinical data, the overexpression of *ACTL6A* and *BRD9* reduced the survival time of patients in a set of cancers. These results suggest that the amplification of *BRD9* and *ACTL6A* could be a universal mechanism in human cancer and may play critical roles in tumor formation. Additionally, we found that both the oxidative phosphorylation pathway and ribosome pathway were downregulated by the inhibition of *ACTL6A* or *BRD9*. However, the specific mechanism of the oncogenic processes remains unclear. In addition, a significant comutation relationship between *ACTL6A* and *BRD9* was observed in our research (p< 0.001, log odds ratio = 1.051). From the above findings, both *ACTL6A* and *BRD9* remarkably affected the same biological pathways. Moreover, a general comutation relationship exists between *ACTL6A* and *BRD9*; however, it is still unknown whether a functional interaction exists between them.

Studies have revealed that ACTL6A is oncogenic in human cancer [12–13]. Some studies have illustrated that overexpressed ACTL6A promotes epithelial-mesenchymal transition (EMT) and invasion in multiple cancers, such as osteosarcoma, hepatocellular carcinoma, and glioma [14–16]. Thus, ACTL6A could function as a new prognostic indicator. Recently, studies have shown that high-level ACTL6A plays an essential role in stemness maintenance. Moreover, ATP produced by mitochondria affects stem cell pluripotency through ACTL6A in embryonic development [15–17]. This suggests that ACTL6A plays key roles in embryonic development and is “reactivated” during tumor formation. Thus, we hypothesized that the aberrant overexpression of *ACTL6A* might reactivate some signaling pathways that occur during embryonic development and drive the oncogenic process. These processes maintained the stemness state of the cell and further promoted EMT. Moreover, previous studies have shown that *ACTL6A* is coamplified with *TP63* and may induce regenerative proliferation through the activation of YAP/TAZ in squamous cell carcinoma and glioma [8, 18]. In this study, we discovered a universal comutation relationship between *ACTL6A* and *TP63* across almost 25 cancers (p<0.001, log odds ratio>3). This comutation relationship is likely to be a common condition in human cancers. However, whether the activation of the YAP/TAZ pathway is also the main oncogenic mechanism in other cancers is not clear.

For *BRD9*, we indicated remarkable copy number amplifications across multiple human cancers. Moreover, we also discovered that the increased expression level of BRD9 leads to worse survival based on clinical data. The amplification of *BRD9* may play a significant role in cancer. However, as a newly discovered subunit in the SWI/SNF complex, the precise function of *BRD9* is still elusive. The study of proteomics illustrated that GBAF (a non-canonical GLTSCR1L- and BRD9-containing SWI/SNF complex, shown in Fig 1), is an enhancer-associated chromatin remodeler [19]. It binds to the specific enhancer through GLTSCR1L, and add the acetyl to the 27^th^ lysine of the core histone H3 (H3K27AC) [19]. The acetylation of histone remodels the chromatin and regulate the gene transcription [19]. BRD9 plays key role in this process. After inhibiting bromodomain of BRD9, GLTSCR1L dislocation from its binding site at H3K27ac-associated enhancer [19]. This leads to the genome-wide downregulating of enhancer transcription [19]. Therefore, perhaps the overexpression of BRD9 lead to more interaction between GBAF and enhancer of genome, which promote the transcription of some oncogenic genes, leading to the formation of cancer. As for the downstream pathways of BRD9, a recently study for leukemia reported that *BRD9* promote the survival of AML cells mainly via *STAT5* activation [20]. While the researcher also indicated that BRD9 may show a cell type-specific genomic binding pattern. This may explain the different pathways enriched in different BRD9 KD cell lines. Interestingly, according to previous pathway analysis for additional 3 Geo datasets for BRD9, results indicated that the depletion of BRD9 can affect the ribosome pathway and oxidative phosphorylation pathway in different cell lines. However, because of lacking related experiments, the specific mechanism of BRD9 in other cancers is still uncovered. Therefore, further research is needed in future. Recent research has indicated that GBAF (also called ncBAF) regulates naïve pluripotency in embryonic stem cells and highly cooperates with *BRD4*, another member of the BET family [21]. Therefore, we hypothesized that the overexpression of BRD9 might also receive signaling from embryonic stem cells and induce a metastatic stem-like profile in cancer cells.

As for the selection of potential treatment targets, a research published last year may provide some clues for us. In this research, in order to predict which genes are required for tumor survival and discover the vulnerabilities of cancer, 501 genome-scale RNAi screens were performed in diverse human cancer cell lines (http://depmap.org/rnai) [22]. According to the result, *ACTL6A* is “common essential” for at least 90% cancer lines, however, there is still no any druggable structure of ACTL6A reported [22]. As for *BRD9*, it has shown a “strongly selective” of dependency for these cell lines, which means that some specific cancer types are strongly depended on this gene, such as synovial sarcoma (p value = 2.9e-04) and acute lymphoblastic leukemia (p value = 1.4e-05) [22]. Besides, the druggable structure and bioactive compounds of BRD9 have been discovered so far. Increasing small-molecule inhibitors of bromodomain-containing protein, such as GSK2801 and BI9564, have been discovered in the past decade [23, 24]. Moreover, more and more studies suggested that BRD9 plays key roles in the proliferation of cancers. For example, a recently research already indicated that BRD9 maintains proliferation of AML cells, and the application of GSK2801 repressed the proliferation of AML[20]. Another study demonstrated that the inhibition of BRD9 acted in a synthetic lethal manner in cBAF-deficient cancer, and the inhibitor of BRD9 effectively decreased the proliferation of cancer cells in synovial sarcoma and malignant rhabdoid tumors [25]. All of these findings highlight the expansive potential of BRD9 as a therapeutic target in multiple human cancers. Therefore, compared to ACTL6A, BRD9 could be a better choice for us to develop the treatment of cancers.

## Conclusions

Above all, we first indicated the common amplification of *BRD9* and *ACTL6A* in cancers, which could be regarded as potential treatment targets in the future. Although we provided some clues to explain their oncogenic roles in human cancers, further analysis is still needed to understand the specific mechanisms that occur during these processes.

## Supporting information

S1 Fig The genetic alteration frequency of SWI/SNF complex in 25 cancers. (PDF)

S1 Table The mutation frequency of each subunit within SWI/SNF complex in 25 cancers. (XLSX)

S2 Table The chi-square test analysis for ACTL6A and BRD9 protein data within ovarian cancer. (XLSX)

S3 Table The overlapping genes of ACTL6A and BRD9 knockdown. (XLSX)

S4 Table mRNA changes for the affected genes of these two pathways across 31 cancer types. (XLSX)

S5 Table Summarized associated pathways for additional 3 GEO datasets BRD9 knockdown. (XLSX)

S6 Table Co-mutation relationship among subunits of SWI/SNF complex. (XLSX)

S7 Table The mutation and copy number alteration frequency of SWI/SNF complex within 25 cancer types. (XLSX)

S8 Table Specific subunits of SWI/SNF complex with mutation frequency more than 10% in certain cancers. (XLSX)
