# Supplementary figures and images for: The genetic alteration spectrum of the SWI/SNF complex: The oncogenic roles of BRD9 and ACTL6A
Source: PLoS One. 2019 Sep 10;14(9):e0222305. doi: 10.1371/journal.pone.0222305 (PMC6736241; doi:10.1371/journal.pone.0222305)

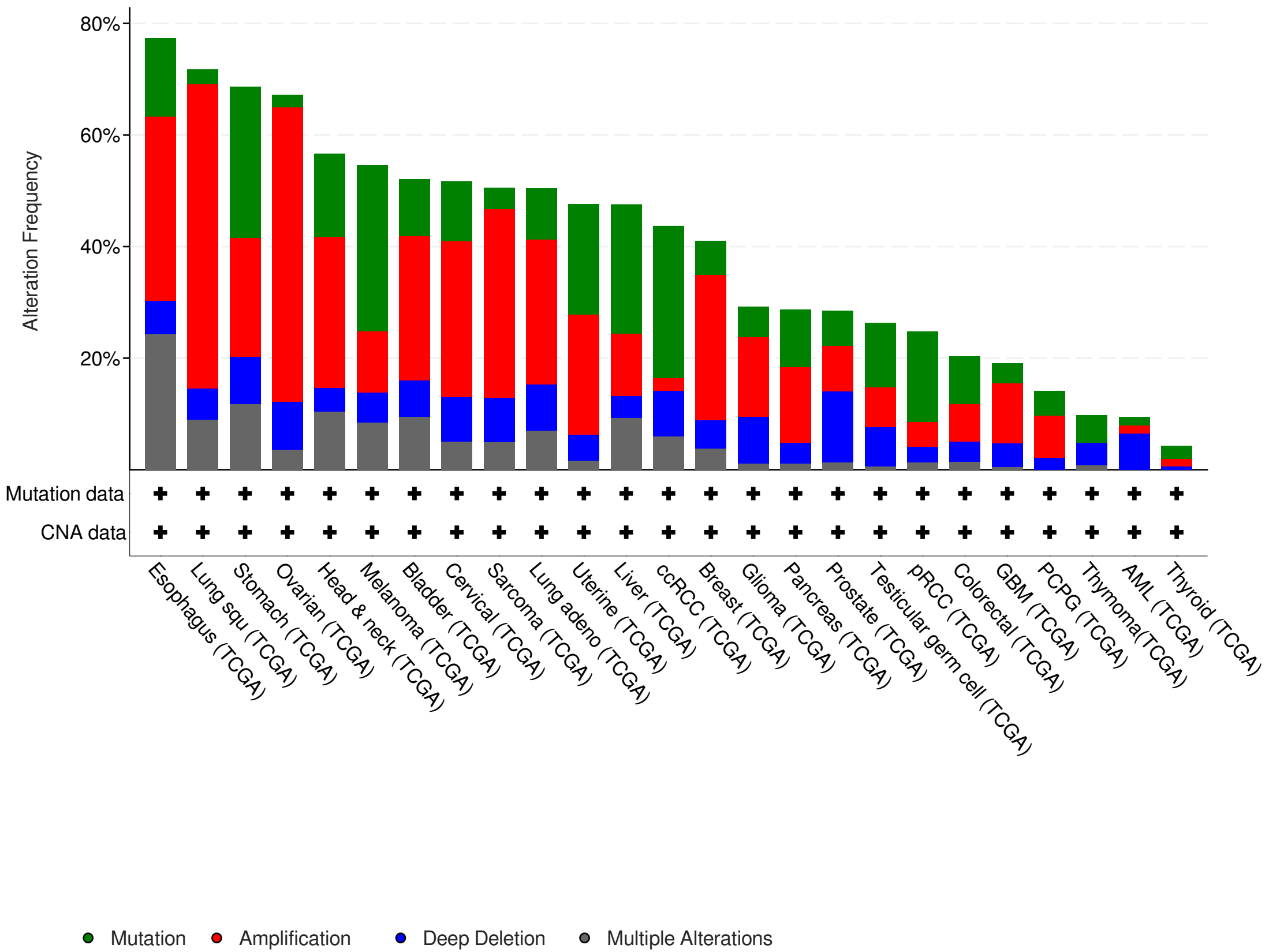

Supplement: S1 Fig — (PDF) [file pone.0222305.s001.pdf]
